# Brain Network Alterations in Alzheimer's Disease Identified by Early-Phase PIB-PET

**DOI:** 10.1155/2018/6830105

**Published:** 2018-01-08

**Authors:** Liping Fu, Linwen Liu, Jinming Zhang, Baixuan Xu, Yong Fan, Jiahe Tian

**Affiliations:** ^1^Department of Nuclear Medicine, General Hospital of the Chinese People's Liberation Army, 28 Fuxing Rd, Beijing, China; ^2^National Laboratory of Pattern Recognition, Institute of Automation, Chinese Academy of Sciences, 95 Zhongguancun East Road, Beijing, China; ^3^Department of Radiology, Perelman School of Medicine, University of Pennsylvania, Philadelphia, PA 19104, USA

## Abstract

The aim of this study was to identify the brain networks from early-phase ^11^C-PIB (perfusion PIB, pPIB) data and to compare the brain networks of patients with differentiating Alzheimer's disease (AD) with cognitively normal subjects (CN) and of mild cognitively impaired patients (MCI) with CN. Forty participants (14 CN, 12 MCI, and 14 AD) underwent ^11^C-PIB and ^18^F-FDG PET/CT scans. Parallel independent component analysis (pICA) was used to identify correlated brain networks from the ^11^C-pPIB and ^18^F-FDG data, and a two-sample* t*-test was used to evaluate group differences in the corrected brain networks between AD and CN, and between MCI and CN. Our study identified a brain network of perfusion (early-phase ^11^C-PIB) that highly correlated with a glucose metabolism (^18^F-FDG) brain network and colocalized with the default mode network (DMN) in an AD-specific neurodegenerative cohort. Particularly, decreased ^18^F-FDG uptake correlated with a decreased regional cerebral blood flow in the frontal, parietal, and temporal regions of the DMN. The group comparisons revealed similar spatial patterns of the brain networks derived from the ^11^C-pPIB and ^18^F-FDG data. Our findings indicate that ^11^C-pPIB derived from the early-phase ^11^C-PIB could provide complementary information for ^18^F-FDG examination in AD.

## 1. Introduction

The current diagnostic criteria for Alzheimer's disease (AD) include amyloid-*β* (A*β*) and fludeoxyglucose F 18 (^18^F-FDG) positron emission tomography (PET) imaging biomarkers that provide amyloid burden and neuronal injury information [1]. Under both physiological and pathological conditions, the cerebral blood flow is coupled to cerebral metabolic rates of glucose measured by FDG-PET [2, 3]. Several studies have reported that perfusion data estimated from early-phase (^11^C)-labeled Pittsburgh Compound B (^11^C-PIB), referred to as perfusion PIB (^11^C-pPIB), correlated with glucose metabolism as estimated by ^18^F-FDG [4–6]. Moreover, recent PET studies using amyloid and Tau tracers indicated that early-phase images of PET tracers provided information on brain perfusion, closely related to glucose metabolism, and could be used as a neurofunctional biomarker [7–9]. Previous studies mostly focused on regions of interest or on whole brain voxel wise measurements [5, 6, 10], which are not equipped to capture distributed variations in cross-brain networks. It remains unclear how early-phase PIB-PET and glucose metabolism correlate with each other and how such a relationship varies with disease progression at the brain network level.

Nowadays, multivariate statistical paradigms (e.g., principal component analysis [PCA] or independent component analysis [ICA]), which assess distributed variations and their interrelationships in multiple neuroimaging data, provide a better framework for the integrative analysis of multimodal imaging data. As a data-driven analytic method, ICA is a powerful tool to investigate brain networks based on neuroimaging data. This data can be collected with, including functional magnetic resonance imaging (fMRI) [11], magnetoencephalography [12], electroencephalography [13, 14], and structural MRI [15] and PET imaging [16]. Parallel independent component analysis (pICA) [17] is a variation of ICA that allows one to estimate independent components as well as multimodal patterns or mixed coefficients. pICA has recently been used to study the mechanisms by which A*β* deposition leads to neurodegeneration and cognitive decline [18]. It was also used to study the spatial patterns of A*β* deposition and glucose metabolism across an AD population [19]. Moreover, pICA has been used successfully to reveal the complex relationship between different PET elements of AD pathophysiology [19]. pICA, therefore, promises to be a suitable means of exploring the spatial patterns of regional cerebral blood flow (rCBF), evaluated by pPIB, and glucose metabolism at the level of the whole brain network.

In the present study, we adopted pICA to derive brain networks from early-phase of ^11^C-PIB and ^18^F-FDG to explore their relationships across AD, mild cognitive impairment (MCI), and cognitively normal (CN) patient groups. This study was designed to (1) identify whether distinctive functional connectivity networks, such as the default mode network (DMN), can be detected from early-phase ^11^C-PIB data and (2) to explore the discriminability of the network derived from ^11^C-pPIB for distinguishing AD/MCI from CN.

## 2. Results

### 2.1. Patient Characteristics

As described in our previous study [10], patients in the MCI groups (*F* = 4.23, *p* = 0.02) were older than in the CN and AD groups, and there was no significant difference in gender or the level of education for the two groups (*p* = 0.13 and 0.52, resp.). Cognitive performance, estimated from Clinical Dementia Rating (CDR) and Mini-Mental State Examination (MMSE) tests, was significantly worse in AD patients than MCI and CN participants (*F* = 65.93, *p* < 0.001). However, no significant difference in MMSE test results was observed between the MCI and CN groups (Table 1). Furthermore, the amyloid status of all participants is shown in Table 1; the cerebellar gray matter was used as the reference region and the standard uptake value ratio (SUVR) cutoff value was set to 1.15 [20, 21].

### 2.2. Correlated ^11^C-pPIB and ^18^F-FDG Networks

**Figure 1:**
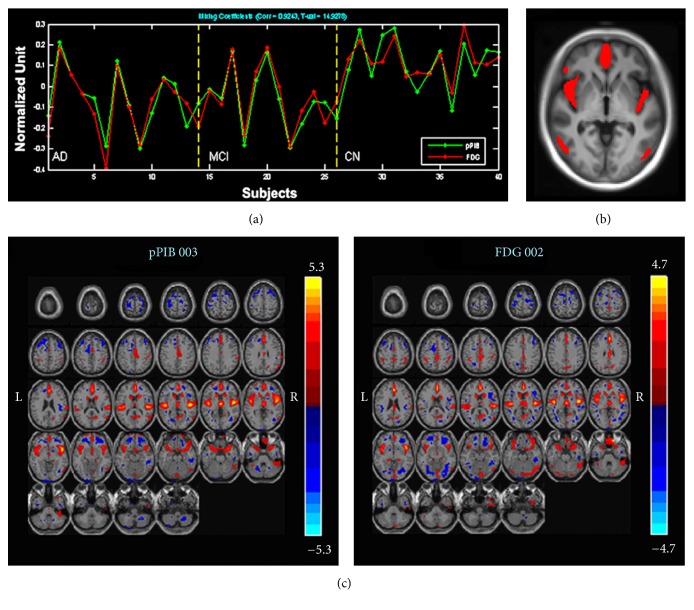
Correlated components of ^11^C-pPIB and ^18^F-FDG. (a) Loading parameters with a significant correlation in all participants with ^18^F-FDG (red) and ^11^C-pPIB (green). (b) Common regions of the correlated ^18^F-FDG and ^11^C-pPIB components, including bilateral medial frontal gyrus, temporal lobe, and right insular lobe. (c) Correlated components of ^11^C-pPIB (left) and ^18^F-FDG (right), including the medial frontal gyrus, anterior cingulate cortex, posterior cingulate cortex/precuneus, superior temporal gyrus, temporal pole, and orbitofrontal gyrus. ^11^C-pPIB, (^11^C)-labeled Pittsburgh Compound B; ^18^F-FDG, fludeoxyglucose F 18.

One pair of components (networks) was identified. It had the highest correlation (*R* = 0.92) between the ^18^F-FDG and ^11^C-pPIB data and was largely colocalized with the DMN [22, 23]. As listed in Table 2, the highest correlated component pair also differed significantly between AD/MCI and CN in their loading coefficients. This pair of components showed that a decrease in ^18^F-FDG uptake correlated with a decrease in perfusion in the frontal, parietal, and temporal regions, including the medial frontal gyrus (MFG), anterior cingulate cortex (ACC), posterior cingulate cortex (PCC)/precuneus, superior temporal gyrus (STG), temporal pole, and orbitofrontal gyrus (Figure 1).

### 2.3. Group Comparisons of ^18^F-FDG and ^11^C-pPIB within the Correlated Network

**Figure 2:**
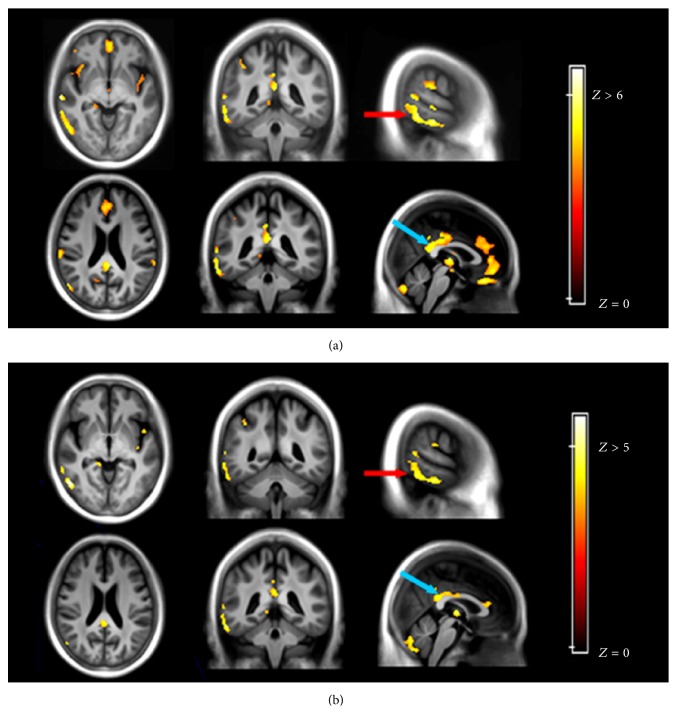
Comparison of ^18^F-FDG (a) and ^11^C-pPIB (b) between Alzheimer's disease (AD) and cognitively normal (CN) groups. Images show a similar decrease in the radioactive pattern, such as for the right superior temporal gyrus (red arrow, *x* = −50, *y* = −4, *z* = 5) and posterior cingulate cortex (blue arrow, *x* = 2, *y* = −41, *z* = 23). ^11^C-pPIB, (^11^C)-labeled Pittsburgh Compound B; ^18^F-FDG, fludeoxyglucose F 18.

Tables 3 and 4 summarize the group differences within the correlated networks in ^18^F-FDG and ^11^C-pPIB measurements between AD and CN groups (Figure 2). Despite fewer regions detected by ^11^C-pPIB than ^18^F-FDG in the intergroup comparisons, similar patterns were observed. The hypometabolic regions largely colocalized with the hypoperfusion areas, including the STG, Limbic lobe/ParaHippo, superior parietal lobe (SPL), PCC, and ACC.

**Figure 3:**
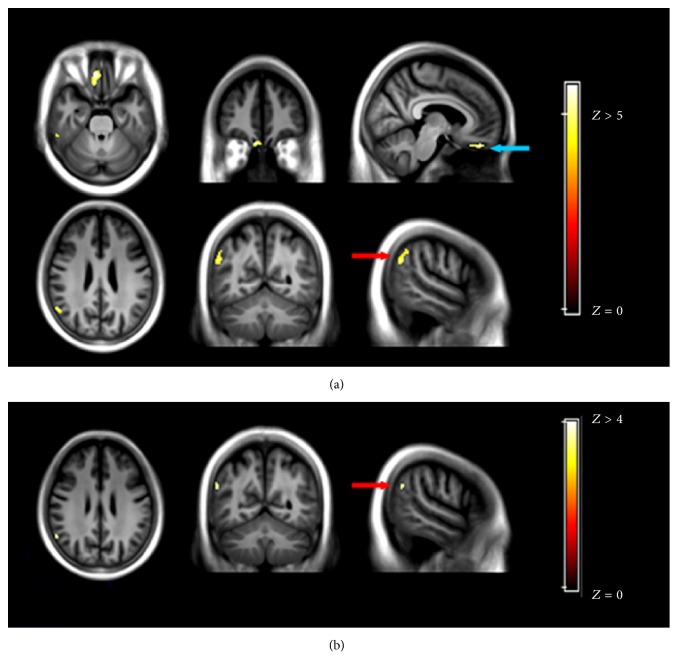
Comparison of ^18^F-FDG (a) and ^11^C-pPIB (b) between mild cognitive impairment (MCI) and cognitively normal (CN) groups. (a) ^18^F-FDG images showing hypometabolic regions in the rectal gyrus (blue arrow, *x* = 8, *y* = 42, *z* = −26) and inferior parietal lobule (red arrow, *x* = 54, *y* = −52, *z* = 50) and (b) ^11^C-pPIB, showing only hypoperfusion in the inferior parietal lobule (red arrow, *x* = 58, *y* = −56, *z* = 46). ^11^C-pPIB, (^11^C)-labeled Pittsburgh Compound B; ^18^F-FDG, fludeoxyglucose F 18.

The comparison between MCI and CN patients within the correlated network revealed that the ^18^F-FDG uptake was less in the rectal gyrus/Brodmann area 11 (BA11), BA40, left PCC, BA20, and inferior parietal lobule (IPL)/STG (Figure 3). In contrast, hypoperfusion was only detected in the IPL.

No statistically significant differences were observed in ^18^F-FDG or pPIB data for AD and MCI patients.

## 3. Discussion

In contrast to earlier studies looking into the dual-features of dynamic PIB-PET and the similarities between ^11^C-pPIB and ^18^F-FDG images [4–6, 10, 24, 25], the present study identified functional brain networks from early-phase ^11^C-PIB data. We first used pICA to identify the brain networks from the ^11^C-pPIB-PET imaging data and then explored the discriminability of the brain networks in diagnostic group differences. Concomitantly we were able to evaluate the use of ^11^C-pPIB as a neurofunctional biomarker for AD.

### 3.1. Highly Correlated Networks of ^11^C-pPIB and ^18^F-FDG

It is well-documented that there are changes in the brain structure, function, and cognition in AD patients associated with changes in brain networks [26–29]. Using resting state functional connectivity MRI (rs-fMRI), both intra- and internetwork correlations have already been detected in AD patients. These mainly involved DMN, dorsal attention, salience, control, and sensory-motor networks [29, 30]. Although AD is associated with widespread disruption of functional connectivity, the DMN is generally affected the most. Specifically, a declined functional connectivity and hypometabolism were observed consistently using various methodologies [27–29]. In the present study, highly correlated brain networks of ^11^C-pPIB and ^18^F-FDG data were identified using pICA. The correlated networks of ^18^F-FDG and ^11^C-pPIB covered the MFG, ACC, PCC/precuneus, STG, temporal pole, and orbitofrontal gyrus and largely colocalized with the DMN [22, 23]. The DMN regions are active at rest (hence the term “default”) [22] but are less active during demanding cognitive tasks. Under physiological conditions, up to 80% of the entire energy consumption by the brain at rest is spent on glutamate cycling, a biochemical process that can be observed by FDG-PET [31, 32]. In fact, the DMN is roughly divided into three major subdivisions, each with its own functional property: the ventral medial prefrontal cortex (supports emotional processing); the dorsal medial prefrontal cortex (self-referential mental activity); and the posterior cingulate cortex and adjacent precuneus plus the lateral parietal cortex (the recollection of prior experiences). These functional properties of DMN can be affected during task performance and also by various diseases [33]. For example, the episodic memory, requiring functional connectivity within the DMN [34, 35], is impaired in the early stages of AD. The abnormalities of DMN functional connectivity worsen with disease progression and are believed to explain the hypometabolism found in PET studies [36–39]. Moreover, FDG-PET measures both the CBF and the neuronal and synaptic activity [40]. A decrease in CBF is, furthermore, an indirect indicator of impairment caused by a decrease in the demand for blood. Nevertheless, the highly colocalized brain networks between glucose metabolism/rCBF and functional connectivity at rest indicated that glucose consumption and changes in rCBF are coupled and underpinning the neural activity. It is therefore argued that ^11^C-pPIB can be used in the future as a neurofunctional biomarker for neuroscience research.

### 3.2. Group Comparison of ^11^C-pPIB and ^18^F-FDG Measurements in Correlated Brain Networks

The second goal of this study was to evaluate whether the brain networks derived from ^11^C-pPIB data could differentiate AD/MCI patient groups from CN groups. Consistent with previous reports, AD patients were characteristically hypometabolic in the medial temporal lobe, STG, ACC, PCC/precuneus, SPL, and lateral temporoparietal cortex [36, 39]. ^11^C-pPIB also revealed significant intergroup differences, with regions of hypoperfusion in the STG, inferior temporal gyrus, SPL, and PCC, which is consistent with previous studies using ^99m^Technetium-single photon emission computed tomography showing specific patterns of hypoperfusion in parietal-temporal cortical areas [41, 42]. The comparison between MCI and CN patients revealed hypometabolism in the rectal gyrus/BA11, IPL, left PCC, BA20, and IPL/STG, but only the IPL was detected by ^11^C-pPIB. The most reliable, early changes in metabolism are believed to be seen in the PCC [24, 36]. In fact, the IPL is an important region of the DMN and has a close functional connection with the PCC/precuneus [43, 44]. Esposito et al. reported that MCI patients who convert to AD showed increased connectivity in the right IPL, suggesting that this region plays an active role in the AD process [45]. Arguably, the hypometabolism and hyperperfusion that was detected in the IPL in this study was indicative of early neurodegeneration in AD. No significant difference was detected by either ^18^F-FDG or ^11^C-pPIB when comparing AD and MCI groups in the current study. We support our observations as follows. Firstly, the AD patients enrolled in the study who received the dual-tracer PET scan had slight to mild dementia. In addition, despite the amnestic type of the recruited MCI patients, MCI is still a heterogeneous syndrome and subject to various pathological substrates and clinical progress [46]. Therefore, the ^18^F-FDG and ^11^C-pPIB data may not detect a difference between AD and MCI due to the differences in clinical symptom severity and the heterogeneity in MCI. Secondly, the MCI patients were significantly older than the AD patients. Although age was regressed in the data analysis and no obvious vascular diseases were found on MR images of all subjects, the CBF will be affected by atherosclerosis, which usually progresses with aging. Thus, both patient's age and its possibly associated reduced CBF may have obscured our results. As discussed, the brain network of ^11^C-pPIB was found highly colocalized with DMN in AD pathology in this study. It is argued that disease-specific alternations of the brain networks can be detected by ^11^C-pPIB corresponding to distinctive pathophysiological processes, which merits further studies.

One limitation of the present study is the relatively small sample size. More recruits would give a larger dataset, which might help to identify the fine-grained brain networks underpinning ^11^C-pPIB and ^18^F-FDG. Furthermore, only the typical AD and amnestic MCI patients were enrolled in the current study and their disrupted brain networks mainly involved the DMN [27, 28, 47]. However, AD patients with atypical symptoms, such as Logopenic primary progressive aphasia and posterior cortical atrophy, had language and visual brain network dysfunctions matching their clinical symptoms [19, 47]. ^11^C-pPIB might, therefore, be used to validate the syndrome-specific alterations of the brain network in these clinical variants of AD. In addition, the present study did not investigate the functional networks derived from rs-fMRI data of the same cohort. Therefore, it is impossible to directly correlate the networks of ^11^C-pPIB and ^18^F-FDG with the functional networks in rs-fMRI data.

## 4. Conclusions

Here, we explored the ^11^C-pPIB spatial distribution pattern in AD, MCI, and CN patients. The pICA results revealed that the hypoperfusion pattern detected by ^11^C-pPIB was in agreement with the hypometabolism reflected by ^18^F-FDG, both of which were colocalized with the DMN. These results validated that ^11^C-pPIB could be a reliable biomarker of neural function and provide complementary information for ^18^F-FDG examination in AD.

## 5. Materials and Methods

### 5.1. Eligibility and Study Design

The study cohort was the same as in our previous studies [10, 25] and included 14 AD, 12 MCI, and 14 CN patients. The study was approved by the institutional review board of the Chinese PLA General Hospital. The study was compliant with the principles of the Declaration of Helsinki. All participants, or their appropriate representatives, signed informed consent forms after receiving a comprehensive written and verbal description of the study.

### 5.2. PET/CT Imaging

All patients underwent ^11^C-PIB and ^18^F-FDG scanning in a random order within two weeks. PET/CT scanning was performed using a Biograph Truepoint 64 (Siemens Healthcare, Germany) consisting of a PET scanner and a multislice CT. A vacuum cushion was used to restrict the participant's head to minimize movement during the scanning.

^11^C-PIB was synthesized from its corresponding precursors as described elsewhere [48]. In brief, ^11^C-PIB was synthesized by bubbling the ^11^CH3-Triflate through an acetone solution of 6-OH-BTA-0. It was then purified by semipreparative HPLC and reformulated with a radiochemical purity of >95% and a specific activity of 50 GBq*µ*mol^−1^ (1.48 Ci*µ*mol^−1^). The protocol for ^11^C-PIB scanning included an initial CT acquisition with intravenous tracer injection, followed by an immediate dynamic PET scan. A spiral CT for the brain was acquired with CT parameters of 120 kV, 100 mA, and slice thickness 3.75 mm, equal to that of PET. Then, a dynamic PET emission scan in 3D acquisition mode was started simultaneously with a single intravenous bolus of ^11^C-PIB at 4.81–5.55 MBq (0.13–0.15 mCi) kg^−1^. Dynamic brain PET images were collected continuously for 60 min, and the data were binned into 26 frames (1 × 10 sec, 6 × 5 sec, 4 × 20 sec, 2 × 1 min, 3 × 2 min, and 10 × 5 min).

^18^F-FDG-PET/CT scans were obtained 50 min after an intravenous injection of ^18^F-FDG at 4.81 to 5.55 MBq (0.13–0.15 mCi) kg^−1^. All participants were instructed to fast for 4 to 6 h. The blood glucose levels were also measured before injection to ensure that the levels were within the reference range. A 5-min frame was collected in 3D acquisition mode. Data obtained from the CT scans were used to correct the attenuation for PET emission data.

### 5.3. MR Structural Imaging

All participants underwent structural MRI with a 3.0-T GE scanner (Signa HD, WI, USA) and a standard GE quadrature head coil. The MRI and PET/CT examinations were performed within one week. The scan protocol included a high-resolution 3D T1-weighted spoiled gradient recalled echo sequence (TR = 7.0 ms, TE = 2.9 ms, Inversion time = 450 ms, thickness = 1.2 mm, matrix = 256 × 256, FOV = 240 mm, and in plane resolution = 0.9 × 0.9 mm^2^) to produce contiguous sagittal anatomic images for subsequent spatial normalization and coregistration.

The preprocessing of MRI and PET imaging is detailed elsewhere [10]. Specifically, all structural MRI images were segmented into gray matter, white matter, and cerebrospinal fluid and then used to construct a population template using DARTEL of SPM8 (http://www.fil.ion.ucl.ac.uk/spm). The mid-frame (the 16th frame) of the dynamic PIB images and FDG images was coregistered with the corresponding MRI scan, and the PET scans were transformed to the population template with the deformation fields generated in the registration procedure of the MRI scans. Finally, all images were spatially normalized to the Montreal Neurological Institute space.

### 5.4. Computation of ^11^C-pPIB Image from Dynamic ^11^C-PIB Scans

The procedure to compute the ^11^C-pPIB images from the dynamic PIB scans has been reported previously [10]. Particularly, ^11^C-pPIB images were computed based on a 7-min time-window of the dynamic ^11^C-PIB-PET, starting from 9th frame to 15th frame and corresponding to the frames of 1.33–8 min, yielding the highest correlation between ^18^F-FDG and ^11^C-PIB (*R* = 0.87). Also, ^11^C-PIB and ^18^F-FDG images shared a similar radioactive distribution pattern in CN, MCI, and AD groups.

### 5.5. Statistical Analysis

The pICA algorithm (Fusion ICA Toolbox, http://icatb.sourceforge.net, with MATLAB 7.1) was applied to the ^11^C-pPIB and 18F-FDG images of all the patients to jointly extract statistical factors from the 11C-pPIB and 18F-FDG data and identify their mutual relationship.

The number of independent components was set to eight, based on a previous study [49]. The outputs from the pICA were pairs of ^11^C-pPIB and ^18^F-FDG independent components. Their correlation coefficients were indicative of a relationship between the two modalities. The independent components of ^11^C-pPIB and ^18^F-FDG data measured the perfusion and metabolic variability among the participants. All components had a threshold at a supra level, |*Z*| > 1.5, to identify statistically significant regions that contributed to the overall signal of the corresponding components. Correlation coefficients between components of the two imaging modalities were used to identify the most associated pairs of components.

Subsequently, voxel wise ^11^C-pPIB and ^18^F-FDG measurements within the most associated pair of components were compared between AD-CN, MCI-CN, and AD-MCI groups using a two-sample *t*-test. The multiple comparisons were corrected for using the AlphaSim program in REST (http://restfmri.net/forum/rest) with a full-width at half-maximum of 6 mm. The threshold for the group differences was *p* < 0.05 with the AlphaSim correction (with *p* < 0.001 threshold and a minimum cluster size of 12 voxels).

## Figures and Tables

**Table 1:** Demographic, clinical, and neuropsychological characteristics of the patients.

	AD (*n* = 14)	MCI (*n* = 12)	CN (*n* = 14)	*p* value
Sex (male/female)	4/10	8/4	5/9	0.13
Age (years)	68.1 ± 9.9	75.8 ± 8.6	67.4 ± 5.0	0.02^*∗*^
Education (years)	11.6 ± 3.9	13.3 ± 3.8	11.9 ± 4.1	0.52
Amyloid status (PIB +/−)	12/2	9/3	1/13	<0.001^*∗*^
MMSE	19.4 ± 3.3	27.3 ± 1.6	28.4 ± 1.2	<0.001^*∗*^
CDR	0.96 ± 0.13	0.5	0	<0.001^*∗*^

With the cerebellar cortex as the reference region, voxel wise semiquantitative calculations of global standardized uptake value ratios (SUVR) for all subjects were performed. The amyloid status was reflected by the Pittsburgh Compound B (PIB) burden and the cutoff value of SUVR was set to 1.15 to determine PIB positive or negative. Chi-square was used for the gender comparison; one-way ANOVA with a Bonferroni post hoc test was used for age and neuropsychological test comparisons. ^*∗*^*p*< 0.05. AD: Alzheimer's disease; MCI: mild cognitively impaired; CN: cognitively normal; MMSE: Mini-Mental State Examination; CDR: Clinical Dementia Rating.

**Table 2:** Two sample *t*-test results of the highest correlated component pair of ^11^C-pPIB and ^18^F-18F-FDG between AD and CN and between MCI and CN groups.

AD versus CN				MCI versus CN			
^11^C-pPIB		^18^F-FDG		^11^C-pPIB		^18^F-FDG	
*p*value	*T* value	*p*value	*T* value	*p*value	*T *value	*p value*	*T *value
0.0010	−3.6907	0.0002	−4.3774	0.0009	−3.7993	0.0010	−3.7279

AD: Alzheimer's disease; MCI: mild cognitively impaired; CN: cognitively normal; ^11^C-pPIB: (^11^C)-labeled Pittsburgh Compound B; ^18^F-FDG: fludeoxyglucose F 18.

**Table 3:** ^ 18^F-FDG–revealed hypometabolic brain areas differentiating AD and CN groups.

Brain region	Voxel-level			*X*	*Y*	*Z*
	*T*	*Z*	*p*_uncorrected_			
Limbic lobe/R ParaHippo	6.75	5.05	0.000	22	−4	−38
	4.78	3.99	0.000	22	6	−24
R STG/BA39	6.40	4.88	0.000	58	−58	26
	5.18	4.23	0.000	54	−54	44
R MTG/ ITG	5.99	4.68	0.000	54	−64	18
	5.85	4.60	0.000	66	−40	−14
Rectal gyrus	5.89	4.62	0.000	8	32	−26
Limbic lobe/L ParaHippo	5.79	4.57	0.000	−22	4	−22
PCC/BA29	5.64	4.49	0.000	−2	−42	20
	5.51	4.41	0.000	0	−26	32
R MFG/ACC	5.09	4.18	0.000	2	50	−12
	4.88	4.05	0.000	4	38	24
R SPL/BA7	5.01	4.13	0.000	36	−58	54
	4.70	3.94	0.000	40	−48	50
L STG/BA13	4.95	4.10	0.000	−50	−4	0
	4.42	3.76	0.000	−46	6	−2
L STG/BA42/Insula	4.79	4.00	0.000	−64	−36	18
	3.82	3.36	0.000	−54	−32	18
L STG/BA38	4.73	3.96	0.000	26	6	−48
L IFG/BA47	4.70	3.94	0.000	−40	24	−16
	4.09	3.55	0.000	−38	14	−12

Threshold: *T* = 3.45, *p* = 0.001; Extent threshold: *k* = 50 voxel, voxel size: [2.0,2.0,2.0] mm. ^18^F-FDG: fludeoxyglucose F 18; AD: Alzheimer's disease; CN: cognitively normal; STG: superior temporal gyrus; BA: Brodmann area; MTG: middle temporal gyrus; ITG: inferior temporal cortex; PCC: posterior cingulate cortex; MFG: medial frontal gyrus; ACC: anterior cingulate cortex; SPL: superior parietal lobe; IFG: inferior frontal gyrus.

**Table 4:** ^11^C-pPIB–revealed hypoperfusion brain areas differentiating AD and CN groups.

Brain region	Voxel-level			*X*	*Y*	*Z*
	*T*	*Z*	*p*_uncorrected_			
R ITG/BA19	5.66	4.50	0.000	54	−64	−6
	5.32	4.31	0.000	54	−66	14
L STG/BA22	5.00	4.13	0.000	66	−46	6
Limbic lobe/L ParaHippo	4.74	3.97	0.000	−22	4	−22
BA23/PCC	4.41	3.76	0.000	2	−38	24
	4.15	3.59	0.000	−2	−26	30
Insula/BA13	4.39	3.74	0.000	42	−20	12
R STG/BA39	4.37	3.73	0.000	58	−56	26
L STG/BA22	4.18	3.60	0.000	−52	0	0
R ITG/BA20	3.89	3.41	0.000	58	−8	−34
ACC/BA24	3.86	3.38	0.000	2	32	16
Limbic lobe/R ParaHippo	3.84	3.38	0.000	14	−38	−2
R SPL/BA7	3.82	3.35	0.000	36	−58	54
R IPL/BA40	3.81	3.32	0.000	40	−48	44

Threshold: *T* = 3.45, *p* = 0.001; Extent threshold: *k* = 50 voxel, Voxel size: [2.0,2.0,2.0] mm. ^11^C-pPIB: (^11^C)-labeled Pittsburgh Compound B; AD: Alzheimer's disease; CN: cognitively normal; ITG: inferior temporal cortex; STG: superior temporal gyrus; BA: Brodmann area; ACC: anterior cingulate cortex; SPL: superior parietal lobe; IPL: inferior parietal lobule.
